# EMT/MET at the Crossroad of Stemness, Regeneration and Oncogenesis: The Ying-Yang Equilibrium Recapitulated in Cell Spheroids

**DOI:** 10.3390/cancers9080098

**Published:** 2017-07-29

**Authors:** Elvira Forte, Isotta Chimenti, Paolo Rosa, Francesco Angelini, Francesca Pagano, Antonella Calogero, Alessandro Giacomello, Elisa Messina

**Affiliations:** 1 The Jackson Laboratory, Bar Harbor, ME 04609, USA; 2 Department of Medical Surgical Sciences and Biotechnologies, “La Sapienza” University of Rome, 04100 Italy; isotta.chimenti@uniroma1.it (I.C.); p.rosa@uniroma1.it (P.R.); f.angelini@uniroma1.it (F.A.); francesca.pagano@uniroma1.it (F.P.); antonella.calogero@uniroma1.it (A.C.); 3 Department of Molecular Medicine, “La Sapienza” University of Rome, 00195 Roma, Italy; alessandro.giacomello@uniroma1.it; 4 Department of Pediatrics and Infant Neuropsychiatry, “Umberto I” Hospital, “La Sapienza” University of Rome, 00195 Roma, Italy; elisa.messina@uniroma1.it

**Keywords:** spheroids, EMT/MET, TGF-β, EGR-1

## Abstract

The epithelial-to-mesenchymal transition (EMT) is an essential trans-differentiation process, which plays a critical role in embryonic development, wound healing, tissue regeneration, organ fibrosis, and cancer progression. It is the fundamental mechanism by which epithelial cells lose many of their characteristics while acquiring features typical of mesenchymal cells, such as migratory capacity and invasiveness. Depending on the contest, EMT is complemented and balanced by the reverse process, the mesenchymal-to-epithelial transition (MET). In the saving economy of the living organisms, the same (Ying-Yang) tool is integrated as a physiological strategy in embryonic development, as well as in the course of reparative or disease processes, prominently fibrosis, tumor invasion and metastasis. These mechanisms and their related signaling (e.g., TGF-β and BMPs) have been effectively studied in vitro by tissue-derived cell spheroids models. These three-dimensional (3D) cell culture systems, whose phenotype has been shown to be strongly dependent on TGF-β-regulated EMT/MET processes, present the advantage of recapitulating in vitro the hypoxic in vivo micro-environment of tissue stem cell niches and their formation. These spheroids, therefore, nicely reproduce the finely regulated Ying-Yang equilibrium, which, together with other mechanisms, can be determinant in cell fate decisions in many pathophysiological scenarios, such as differentiation, fibrosis, regeneration, and oncogenesis. In this review, current progress in the knowledge of signaling pathways affecting EMT/MET and stemness regulation will be outlined by comparing data obtained from cellular spheroids systems, as ex vivo niches of stem cells derived from normal and tumoral tissues. The mechanistic correspondence in vivo and the possible pharmacological perspective will be also explored, focusing especially on the TGF-β-related networks, as well as others, such as SNAI1, PTEN, and EGR1. This latter, in particular, for its ability to convey multiple types of stimuli into relevant changes of the cell transcriptional program, can be regarded as a heterogeneous "stress-sensor" for EMT-related inducers (growth factor, hypoxia, mechano-stress), and thus as a therapeutic target.

## Highlights:

● EMT/MET play a pivotal role in cell fate decision making for both normal and transformed cells.
● Thus, these mechanisms represent a strategic target for preclinical (basic studies, pharmacologic screening, and biotechnology advances), as well as clinical applications (personalized diagnosis and therapy).
● EMT/MET are finely reproduced within cell spheroid systems, which, as in vitro models of normal and transformed stem cell (SC) niches, represent an adequate cost/benefit biotechnological tool to investigate disease mechanisms, therapeutic targets, and related applications.

## 1. Introduction

Our knowledge of the shared pathways in trans-differentiation processes occurring during organogenesis, post-natal tissue repair/regeneration, and tumorigenesis has greatly expanded in the last decades, thanks also to improvement of in vitro cell culture technologies, namely three-dimensional (3D) tissue-derived spheroid systems. These 3D culture methods have been developed to recapitulate the in vivo growth, differentiation and de-differentiation conditions of normal and cancer cells, by better preserving the biological features of the original source compared to conventional 2D monolayer cultures. In particular, their hypoxic and hierarchical stem cell (SC)-supporting environment favors heterogeneous cell-cell, cell-matrix interactions and cross-talk required to mimic patho-physiological processes. Conversely, these latter are poorly represented in static 2D systems, in which cells are exposed to high O_2_ and nutrient concentrations in the medium, and forced to directly interact with high-stiffness artificial substrates. This artificial condition cannot reproduce the time-course and dose-dependence of specific ligand–receptor interactions and downstream signaling induction. Furthermore, even the orthotopic transplantation of tumor cells, used to define cancer SC features, while representing the gold standard of in vivo experimental models, lacks patient-specific conditions, which are not easy to achieve in the xenograft [1], and it is less expensive and time consuming [1,2].

The reliability of 3D cell spheroid systems has allowed scientists to extend the experimental modeling of normal and malignant SC growth and differentiation. In addition to oxygen gradient, allowing a SC niche-like balance of cell quiescence/proliferation in the spheroid, the specific SC features of drug sensitivity/resistance, as well as phenotypic changes and trans-differentiation ability, can be spontaneously achieved within these systems, opening a window on the natural history of the tissue they came from. Consistently, their use for protocols of in vitro culture of normal and malignant tissue-derived SCs is now available for disease mechanism studies, drug discovery, chemoresistance and high-throughput screening, aiming at identifying molecules that inhibit cancer stem cell (CSC) proliferation, or at modulating tissue-derived stem cells (tSCs) growth and differentiation.

In this perspective, the specific trans-differentiation process of epithelial-to-mesenchymal transition (EMT), which is shared by both normal (developing/regenerating) and neoplastic tissues, can be nicely reproduced within ex vivo cultured spheroids. In these systems, a cell-migration/colonization mechanism is associated with the mechano-sensing apparatus and signaling, characterized by the reversible loss of epithelial (or endothelial) properties coupled with the acquisition of mesenchymal features.

EMT and its opposite MET (mesenchymal-to-epithelial transition) are significantly involved in stemness balance in both normal and malignant cell spheroids, and their modulation is strategic for the achievement of specific cell phenotypes [3,4,5,6]. At molecular level, EMT is mediated by the activation of several transcription factors (TFs), including those belonging to the Snail superfamily, such as SNAI1 and SNAI2 [7], by the loss of cell-junction molecules, such as E-cadherin (encoded by *CDH1*), and the acquisition of mesenchymal markers, such as vimentin. Activation of EMT has been particularly studied in several cancer spheroid models (e.g., mammospheres, prostaspheres, pancreatic spheres, neurospheres from nervous system tumors), as well as in normal tSCs (e.g., cardiospheres, neurospheres, retinal spheres) and embryonic SCs (e.g., blastulation, embryoid bodies, induced cell reprogramming) [8].

The controversial link between EMT and stemness in normal and neoplastic conditions has been extensively highlighted both in vitro and in vivo [9]. While a coexistence of EMT and MET is typical during development and tissue repair, allowing an intermediate phenotype which is possibly associated with stemness features, in cancer the same process is associated with invasion and progression. The two different perspectives postulated by Brabletz [10], in which both the association, as well as the separation between stemness and EMT can occur in cancer development and diffusion (depending on the time-window and microenvironment), may represent more than a realistic hypothesis, which needs to be further tested and exploited in tumor spheroid systems.

In this review, the role of EMT and its reverse process MET in the cell fate decision cross-road will be described, by taking into account the analogies and differences in the same shared signaling pathways, acting as pro-self-renewal in the context of cancer, or as self-growth limiting/differentiating in normal tissues. We will focus specifically on 3D cell spheroids as valuable SC/CSC niche models with a pivotal role in studying the role of EMT/MET related pathways in the modulation of cell stemness, differentiation and trans-differentiation. The key pathway of TGF-β, and its related network will be particularly evidenced, and potential pre-clinical/clinical application highlighted.

## 2. EMT and Stemness in Physiological and Transformed Tissues

EMT and MET processes have been long associated with the balance between stemness and differentiation in multiple cell models (physiologic, pathologic or transformed), with many regulatory pathways involved. This EMT/stemness relationship is often connected to cell ability for spheroid formation and growth, so that spheroid formation is indeed used as a functional SC assay in multiple systems, albeit with some limitations [11]. It is well established that EMT is a finely regulated process involving many interconnected pathways responsible for the phenotypic manifestation of epithelial versus mesenchymal features. A vast amount of data derives from embryology studies, which have identified specific properties modulated during this switch: the basement membrane structure, apical polarity and junctions, motility, and cell adhesion. Different EMT-TFs are responsible for the regulation of these properties, albeit in separate molecular systems, i.e. each cellular activity has its own control circuit made of specific TFs, so that complete EMT requires the simultaneous activation of all of them [12].

Several EMT specific TFs have been associated with stemness phenotypes through several mechanisms, such as modulation of stemness-related miRNAs. One example is the miR-200 family, which comprises members with strong epithelial-promoting effects, while concomitantly targeting multiple stem cell factors, such as Sox2 and Klf4. Zeb1 is an EMT activator which is also able to downregulate the miR-200 family, thus suppressing epithelial transcriptional programs and inducing stemness TFs in both cancer cells and embryonic SCs [13]. Conversely, miR-200c can block the physiological ability of mammary SCs to differentiate into gland tubules. Moreover, miR-200c can also inhibit clonal expansion of both adult and embryonic cancer cells through BMI1 [14], providing an interesting molecular similarity in the EMT-mediated regulation of stemness between normal tSCs and CSCs.

Indeed, pleiotropic proteins are also involved with epigenetic machineries in controlling the EMT and stemness balance. As an example, among its many guardian functions, p53 can be also considered as an “epithelium keeper”, together with members of the miR-200 family, as previously mentioned, which are able to regulate EMT also by inhibiting specific E-cadherin repressors, such as Zeb1 and Zeb2 [15]. It has been shown that decreased p53 and miR-200c levels are associated with promotion of EMT and concomitant increase in the abundance of mammary epithelial and SCs [16].

Another important stemness regulating microRNA is let-7, which has been studied in multiple systems. It is downregulated in fetal neural SCs, and its expression gradually increases during postnatal life and aging, together with p16/p19, promoting the loss of neural SCs [17]. Lin28, an RNA binding protein able to regulate let-7, is also involved in SC function modulation; it is upregulated in both CSCs and induced pluripotent stem cells (iPSs), and its overexpression is able to significantly increase self-renewal and efficiency of reprogramming protocols [18]. Lin28 has been shown to be significantly expressed, particularly in more mesenchymal-like cells, while inducing EMT through let-7 downregulation. Lin28 modulates self-renewal and differentiation of mammary epithelial SCs [19], increases the efficiency of spheroid formation as mammospheres, and promotes migration in breast cancer cells [20].

The relationship between EMT and the regulation of the stemness/differentiation balance emerges also in adult tissues during wound healing when cell cycle re-entry, dedifferentiation (to some extent) and motility are needed for injury repair. In fact, during skin wound healing, basal epithelial cells are required to temporarily suppress their adherent immotile phenotype, migrate towards the wound edges, and then contribute to re-epithelialization. These cells undergo a partial EMT process activated by tissue injury [21], which is necessary for repair, and represent an example of how some dynamic features linked to EMT are required for normal tissue maintenance and healing during post-natal life. Partial EMT during repair occurs in keratinocytes as well, in an EGF/Erk5/SNAI2-regulated way [22]. Albeit historically named after epithelial cells, EGF is able to sustain SNAI2 transcription associated with intermediate EMT states and stemness features. Interestingly, EGF-signaling blocking has been related to impaired stem/progenitor cell functions also in a mesodermal tissue, such as the heart [23]. Moreover, it has been shown that modulation of the β2-adrenergic signaling pathway, which is able to affect the EMT balance in human adult cardiac progenitor cells, is associated with enhanced SC features and increased cell spheroid formation [24].

Partial EMT states associated with stem/progenitor cell functions also derive from the lung, where wound healing is again linked to the acquisition of mesenchymal traits by club and basal cells [25]. These cells are facultative SCs in lung tissue, and when activated by injury, they undergo a transient mesenchymal state, which is necessary for tissue regeneration. They activate the expression of the mesenchymal marker vimentin, while showing mixed epithelial/mesenchymal features. Interestingly, this process, occurring in vivo during tissue regeneration, has also been observed ex vivo in human lung progenitor cells when selectively cultured as spheroids, in association with features of enhanced differentiation potential, as in a SC niche-like microenvironment [26], strenghtnening the modelling potential of cell spheroids ex vivo.

Vimentin is a marker of partial EMT states also in the mammary epithelium. In fact, overexpression of SNAI1 in primary human mammary epithelial cells results in higher vimentin expression levels, with coherently reduced E-cadherin, and these mesenchymal traits are again associated with a higher efficiency of spheroid growth as mammospheres. The same mechanism can be observed in mouse mammary SCs, with mesenchymal features associated with enhanced stemness functions [27]. Moreover, other EMT inducers, such as Six1 and LBX1, can also enhance mammosphere formation through Zeb1 or SNAI1, leading again to SC expansion and mammary hyperplasia in the mouse [28].

EMT circuits have been reported to affect another important SC function, i.e. the balance between symmetric and asymmetric division, which affects the dimension of the SC population itself. In colorectal CSCs, SNAI1 has been shown to promote symmetric division through miR-146a and beta-catenin, thus amplifying the SC pool [29].

Albeit multiple similarities exist in normal versus transformed systems, it has been shown that EMT is activated to different extents and through different TFs in normal mammary epithelial SCs compared to tumor initiating cells [30]. In fact, normal SCs in the basal epithelium rely on a SNAI2-mediated signal for partial EMT activation related to their physiologic support of gland turnover, which is characterized by co-expression of epithelial and mesenchymal markers, including ZEB1 expression. In comparison, mammary tumor initiating cells with properties of CSCs are highly SNAI1 positive and display a stronger mesenchymal phenotype, including complete loss of E-cadherin expression, detachment, acquisition of motility, and lack of ZEB1 expression.

Interestingly, in another important system where the stemness/differentiation balance requires modulation, i.e. cell reprogramming to iPSs, the opposite transition is involved. In fact, fibroblast reprogramming requires MET, which by some authors has been figuratively described as “moving backwards” in their developmental program, reaching towards the more epithelial-like embryonic state. Reprogramming TFs (e.g., Sox2, Oct4, c-Myc, and Klf4) are able to downregulate multiple EMT mediators, such as SNAI1 and TGF-β, and upregulate E-cadherin [31]. Blocking this transition can significantly hamper the efficiency of reprogramming protocols. Moreover, cells at intermediate states during reprogramming closely resemble, at transcriptomic level, MET-driven developmental processes during mesendoderm formation in the primitive streak [32], providing another significant clue linking stemness and EMT.

Considering these examples, it may seem inconsistent that EMT and MET are both associated with the acquisition of stemness features in different systems. The current view is that stemness features are not simply associated with a “more epithelial” or “more mesenchymal” phenotype, but indeed to intermediate so-called “metastable” EMT states [33], which can be encountered during a transition in both directions, and have been studied in both CSCs and development [34]. Studies have compared the molecular, epigenetic, and phenotypic features of trophoblast SCs (brought to intermediate EMT states through MAP3K4 inactivation, or SNAI1 upregulation) to that of invasive breast cancer cells, finding significant similarities between their “metastable” EMT states, both characterized by enhanced stemness functions, such as self-renewal, multi-lineage potential, and motility [35]. Considering the previously mentioned epigenetic control systems, miR-200 can inhibit Lin28, thus linking an intermediate stemness state to a more mesenchymal phenotype, while if let-7 inhibits ZEB, the process is brought towards the opposite end, i.e. towards an epithelial phenotype [30]. Therefore, the Lin28/let-7 ratio seems to play a fundamental role in the balance between transitions, and in the interplay with other mediators, such as SNAI1 and Twist.

It has been proposed that miR-200, Zeb, Lin28 and let-7 are all part of a circuit that modulates the EMT-stemness network through common regulatory factors, which move the activation of stemness features between a more epithelial or more mesenchymal state [36]. This theory of a flexible “stemness window” between EMT and MET may reconcile many different studies that have described apparently contradictory results, highlighting the concept that intermediate states are the ones that may be actually associated with stemness features in both normal and transformed cells.

## 3. EMT-Induced Spheroids as an in Vitro Model of Stem Cell Niches and Tumors

**Figure 1:**
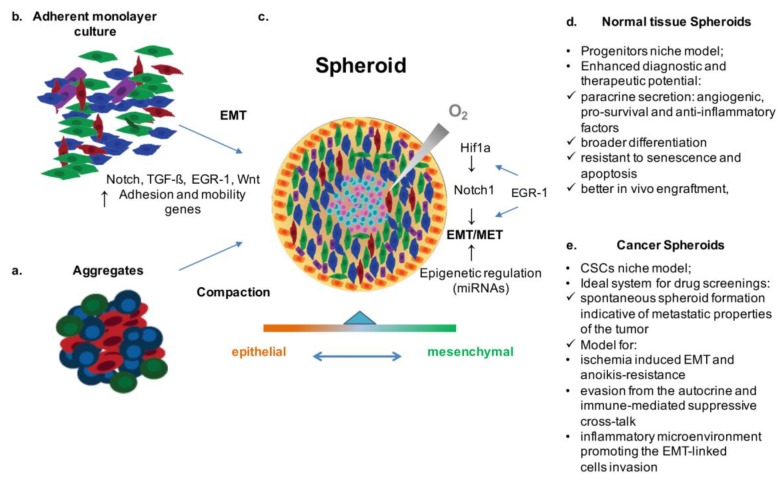
EMT-induced spheroids as an in vitro model of stem cell niches and tumors. Spheroids can be obtained by aggregation and compaction (**a**) or spontaneous formation from monolayer cultures, mediated by activation of EMT related pathway and gene associated with adhesion and motility (**b**). Spheroids are globular and compact structures that can be handled without causing mechanical dissociation of the cells, characterized by a hypoxic core with quiescent cells surrounded by a rim of proliferating cells. The hypoxic gradient activates Notch and other EMT associated pathways favoring the maintenance of a “metastable” state of differentiation within the spheroid (**c**). Characteristics of normal tissue spheroids (**d**) and cancer spheroids (**e**). EMT: epithelial-to-mesenchymal transition; MET: mesenchymal-to-epithelial transition; TGF-β: Transforming Growth Factor-beta; EGR-1: Early growth response protein 1.

As mentioned above, despite the importance of traditional 2D cultures, 3D systems are generally considered as more representative models of living tissues, and have been widely used in stem cell biology, cancer biology, and tissue engineering [37]. In the SC field, spheroids have been obtained from different adult organs, starting from liver [38] and brain [39] over twenty years ago, to various stromal tissues, including human and murine hearts [40], human skeletal muscle [41], human bladder [42], exocrine pancreas [43], thyroid [44], kidney [45], breast [46], lung [26,47], and bone marrow [48]. Compared to monolayer cultures, cells in spheroid cultures are more resistant to senescence and apoptosis, present better in vivo engraftment, enhanced paracrine secretion of angiogenic, pro-survival and anti-inflammatory factors, and a broader differentiation potential [26,38,45,48,49] (Figure 1).

In these conditions, cells are also exposed to more physiological cell-cell, cell-matrix interactions, and recreate a microenvironment which is thought to resemble tissue-resident SC niches [50], characterized by low-oxygen tension, which is important for the maintenance of an undifferentiated phenotype [51]. It has been proposed that low oxygen tension presents a selective advantage for SCs, which are in this way protected from the oxidative stress and DNA damages associated with aerobic metabolism [52]. In particular, oxygen levels seem to modulate the fine balance between proliferation and quiescence in SCs. Hypoxia-mediated stabilization of HIF-1a is known to activate Notch1 pathway, which has been associated with stemness maintenance, but also EMT, and may have a role in maintaining the intermediate “metastable” state previously discussed [53]. One possible drawback of this culture system is that excessive diameter of the spheroid may induce necrosis of the cells in the core. Mechanical dissociation to maintain a small diameter and spinner culture techniques can be adopted to ensure the optimal diffusion of nutrients, oxygen and waste [48,50].

Since the pioneering experiments of Sutherland et al. [54], spheroids have also been widely used in the cancer field, and have proven to be a valuable tool for drug testing, as they allow modeling in vitro the poorly vascularized microenvironment typical of solid tumors, with limited availability of blood-borne oxygen and nutrients. The low oxygen tension within tumors can lead to hypoxia-induced EMT, which is considered a landmark feature of the more aggressive and invasive cancer types [55,56,57,58,59] (Figure 1).

The definition of spheroids however is not always clear. Spheroids are generally described as globular and compact structures, that can be handled without causing mechanical dissociation of the cells, characterized by a hypoxic core with quiescent cells surrounded by a rim of proliferating cells [55]. Nonetheless, aggregates of loosely attached cells are sometimes inaccurately defined as spheroids, even in absence of proper spherical shape, true cell-cell and cell-matrix interactions, and hypoxic gradient [57]. Therefore, it is important to take into account how spheroids have been generated when comparing different studies.

Spheroids can be obtained by aggregation or spontaneous self-assembly. Aggregation can be induced by forcing cell-cell contact through different methods, such as hanging drop, liquid overlay/cell suspension culture, microwell array (round bottom 96 well plates), or microfluidics (i.e. in gel encapsulation) [60]. The initially loose integrins-ECM interaction within these aggregates is followed by E-cadherin accumulation and compaction. Spheroids can also be generated spontaneously from single cells in suspension, budding from monolayers or from adherent cells plated on cationic substrates, such as poly-D-lysine [40,61] or chitosan (the deacetylated derivative of chitin) [62]. Even though spheroids formed by aggregation represent a significant improvement compared to 2D cultures, their gene expression and active pathways are generally different from spontaneously formed clusters, which reflect more physiological mechanisms.

Mesenchymal/epithelial plasticity plays a central role in spontaneous formation of spheroids. Self-assembled human mesenchymal stem cell (MSC) spheroids on chitosan attach and spread on the membranes before retracting their pseudopodia and forming the multicellular spheroids [63]. This process is accompanied by activation of TGF-β, Notch, and Wnt pathways, and upregulation of genes associated with cell adhesion (e.g., integrins) and motility. Similarly, we have shown that TGF-β-mediated EMT is essential for the formation and maintenance of another model of adhesion-dependent spheroid system, that is the cardiosphere: in fact, TGF-β treatment increases cardiosphere formation, while the selective inhibitor SB431542 blocks cardiosphere formation and induces spreading of pre-existing ones [64].

EMT is important also for spontaneous formation of tumoral spheroids, as shown for example by high vimentin and lack of E-cadherin expression in spheroids spontaneously budding from monolayer cultures of ovarian cancer [65,66], which appear to be more clinically relevant models than those obtained by artificial aggregation, and an ideal system for reliable anti-cancer drug screening [67]. The ability to spontaneously form compact spheroids is reflective of an intrinsic molecular program of the parent tumor, and it is a good predictor of its progression, more than the expression of classical mesenchymal markers. While some studies have shown that expression of EMT-inducing TFs, such as Twist, is associated with the acquisition of CSC phenotype and metastatic properties [27,68], others have reported that metastatic tumors do actually retain an epithelial phenotype. Thus EMT may not be essential for the spreading of the primary tumor, but still be involved in the acquisition of chemo-resistance [69,70]. More recently, Beerling et al. [71], through high-resolution cell tracing experiments in a mouse model that spontaneously develops ductal mammary carcinoma, were able to show that the transition to a mesenchymal state is important for cell migration, but does not necessarily confer differential stemness and growth capacity, since most of the migrating cells adopt an epithelial state after the first few cell divisions. Independently from the expression of strictly mesenchymal or epithelial markers, the ability to spontaneously form spheroids is still a good predictor of metastatic potential [72]. This apparent paradox may be explained by the fact that EMT cannot be considered as a unidirectional transition between two very fixed states. As mentioned above, it is indeed a metastable process with different possible intermediate states [9], and, as such, it may not be modelled correctly by depleting or overexpressing classically EMT-associated genes, which may have oncogenic functions independently from their ability to induce EMT [71]. By phenotypic characterization of different ovarian carcinoma cell lines, Huang et al. have identified four different states along the EMT spectrum: epithelial, intermediate epithelial, intermediate mesenchymal, and mesenchymal. Cells with an intermediate mesenchymal phenotype were the most anoikis-resistant and spheroidogenic, suggesting that the most aggressive phenotype is not associated with a fully epithelial or fully mesenchymal state [73]. This Ying-Yang-like equilibrium may represent a crucial point in both normal and transformed cell spheroids in terms of changes in differentiation program and aggressiveness acquirement, respectively.

Indeed, most tumor-derived spheroids (tumorspheres) show a floating appearance, enriched in CSCs. On this basis and with the limitations linked to the method (e.g., high cell density not corresponding to clonal conditions, different spheroids size), several CSC types have been isolated and grown, often allowing the identification of the related targetable signaling pathway [8].

Interestingly, using the same method employed for the respective normal tissue, tumorspheres retaining CSC features have been isolated from brain tumors (e.g., human CD-133+ neurospheres) closely mimicking the genotype, gene-expression profile, and biology of parental tumors. Likewise, non-adherent mammospheres from human normal and transformed mammary epithelial cells have been isolated and extensively studied for their cancer initiating features, tumor heterogeneity, and pharmacologically sensible molecular networks (e.g., mouse breast cancer model of Erb-B2 receptor tyrosine kinase 2 expression and p53-deficiency [74]; study of the Wnt/β-catenin signaling pathway, and Sox2 expression [75]).

The study of colon and ovarian cancer-derived spheroids has also revealed the role of ROCK signaling inhibition in promoting cell survival and propagation, and in the acquisition of stemness features, including expression of CSC markers, capability for differentiation and tumorigenicity [8].

Consistently with their ability to reproduce the natural processes occurring in the normal or transformed tissue they came from, spheroids can also unveil epigenetic/EMT-dependent mechanisms and their related effects. In fact, as post-transcriptional gene expression regulators, several miRNAs participate in modulating self-renewal, differentiation and transformation in normal SCs and CSCs. In a spheroid model of hepatocellular tumor, miR-200a conferred a mesenchymal phenotype to oval-like progenitor cells, including an elongated cell morphology, enhanced cell migration ability, and expression of EMT-representative markers. Furthermore, several CSC-like traits and relative hepatic markers appeared in these cells, exhibiting enhanced spheroid-forming capacity and displaying superior resistance to chemotherapeutic drugs in vitro. All these miR-200-elicited effects occur by targeting the Wnt/β-catenin pathway. In addition, miR-200 participates in epigenetic modulation through a histone deacetylase 4/SP1/miR-200a regulatory network [76].

A very interesting synthesis of the potential critical step in the switch from normal multipotent mammary SCs and tumor initiating mammary CSCs has been addressed by Celia-Terrassa et al. using mammospheres as a 3D spheroid model [77]. In fact, by taking the advantage to reproduce the normal and transformed SC niche, the microenvironment-linked immuno-mediated cross-talk can be easily studied in these systems. With the hypothesis that, as CSCs can do, normal SCs (including mammary) control the immune system (for example down-regulating MHCs complex) to sustain their cellular activity, the authors addressed the role of epigenetic mechanisms, such as miRNAs, and in particular miR-199-a, in promoting both normal and transformed mammary SC properties by repression of their ability (linked to the Ligand-dependent corepressor, LCOR, nuclear receptor) of being sensitized to interferon-induced differentiation and senescence. This epigenetic mechanism, represented by the mir-199-a/LCOR/interferon axis, mediates the evasion from the autocrine and immune microenvironment-mediated suppressive cross-talk, and is conserved in normal SCs and CSCs. These finding may be both mechanistically and pharmacologically strategic, taking also into account that the inflammatory microenvironment can promote EMT-linked cell invasion [77,78,79].

## 4. Discovering Pharmacological Targets in Spheroid Model: The Case of EGR-1/TGF-β Network

Within the molecular networks strategic for cell survival, drug escape and anchorage independence, which have been studied using the spheroid models, the activation of the Early growth response protein 1 (EGR-1) and its downstream signaling components (MAPK/ERK), including its link with TGF-β, represents a milestone in the detection of critical EMT-dependent pharmacological targets. The individual role of these signaling networks, as well as their relationship with the EMT/MET process, are extensively outlined in other reports, including those collected in this issue. Here the role of 3D spheroid models to unravel their function will be better highlighted.

TGF-β signaling has been suggested to have crucial roles in several features of CSCs, such as in tumor initiation, metastasis, and resistance to anticancer drugs [80,81]. As mentioned above, it has also an important role in the spontaneous formation of tumoral spheroids, and in promoting the malignant progression of these structures [81,82].

Among other targets, TGF-β induces EGR-1, which in turn activates the transcription of several mesenchymal proteins, such as type I collagen and TGF-β itself [83,84]. EGR-1 may serve as a target regulated by TGF-β, as mediator for enhanced TGF-β gene expression and target cell responsiveness [85], as well as co-author of physiologic stress response programs [83]. EGR1 is a zinc-finger TF that binds to GC-rich recognition motifs. EGR-1 is also induced by a number of different stimuli, such as anti-cancer drugs, oxidized lipids, hyperglycemia, growth factors and ionizing radiation, and inhibits or stimulates tumor growth depending on the cellular context and the duration of EGR-1 induction [85,86]. While transient induction of EGR-1 is known to activate angiogenesis, sustained EGR-1 expression induces block of angiogenesis, growth arrest, and apoptosis [87]. This TF is able to directly regulate multiple tumor suppressors to induce apoptotic cell death [85,88], including p53 and PTEN. This latter in particular is also strongly related to the ability of CSCs to form spheres, as suggested by experiments where PTEN knockout was potentiating the invasiveness of colorectal cancer spheroidal cells through a 3D extracellular matrix [89].

In addition, EGR-1 is induced by hypoxia and plays a critical role in hypoxia-induced tumor progression, survival, and angiogenesis [90,91]. Thus, 3D spheroids, which model the hypoxic microenvironment of solid tumor, have proven to be a valuable in vitro model to study the dual role of this TF in different contexts. For example, using multicellular tumor spheroids, it has been shown that EGR-1 overexpression makes tumor cells more sensitive to necrosis induced by glucose depletion, and blocking EGR-1 with a shRNA suppresses growth of the tumorspheres [92]. On the other end, in head and neck squamous cell carcinoma, oxytocin treatment significantly reduces cell migration and spheroid formation by upregulating EGR-1 [93].

Interestingly, EGR-1 is also a target of miR-181 involved in TGF-β-mediated tumor mammosphere formation [94,95], and is upregulated in breast cancer cells expressing high level of NF-κB-induced kinase, which has been associated to a more “stemness” phenotype, promoting cancer expansion and mammosphere formation [96]. Silencing of EGR-1 with syntactic catalytic DNA has been reported to inhibit human breast carcinoma proliferation and migration [97], while on the other hand downregulation of gelsolin (indicator of breast cancer) has been correlated with suppression of EGR-1 [98]. In summary, this TF appears to be among the master genes in cellular stress responses. Depending on the cell type, the duration and intensity of the stimuli, EGR-1 can act as a tumor repressor by inducing necrosis/apoptosis, block of angiogenesis and proliferation arrest, or can promote EMT-mediated cell migration, invasion, tumor growth, and acquisition of chemo-resistance [99,100,101].

EMT/MET processes seem to mediate adaptive responses of cancer cells and CSCs to therapy, resulting in poor chemotherapy response and negative prognosis. These developmental programs can be epitomized by oncogenically transformed cells during tumor progression [33,102]. Intriguingly, EMT can trigger reversion to a CSC-like phenotype [27,103], shedding light on a possible association between EMT, CSCs and drug resistance. Moreover, EMT/MET processes are involved in tumor spheroids formation, which have increased resistance to chemotherapeutics compared to 2D cultures, mimicking more closely in vivo tumor behavior. Therefore, standardized high-throughput ex vivo modelling of cancer with 3D cultures derived from patient biopsies can realistically provide a platform for the study of the molecular pathways involved in the evolution of the tumor, and for personalized drug screening and testing, taking into account the enormous variability among patients and within the tumor.

## 5. Conclusions and Future Perspectives

For several tissue-derived cells of heterogeneous origin, grown in an appropriate microenvironment, the spheroid (niche-like)-forming capacity per se is typical of stem/progenitor cells, irrespectively of their normal or neoplastic nature [1,64,104,105]. This spheroid self-building property can be considered as an EMT-dependent process, mediated by TGF-β and its network signaling. The hypoxic gradient within the spheroids might favor the maintenance of a metastable state associated with the acquisition of stem-like features.

Among its many functions, EMT supports migration in tumor (cell evasion and metastasis) and normal cells (embryonic-fetal development and adult tissue repair). Despite all the differences between normal and transformed cells, common mechanisms shared by normal and malignant SCs can be identified, such as those protecting them from suppressive immune cytokine signaling, which can be evidenced in the mammosphere model.

Moreover, spheroids offer an easy experimental tool. As an example, the molecular loops between EGR-1, TGF-β and EMT can be easily studied in 3D models as multicellular spheroid CSC compartments, where this network can be tamed under different stimuli and new drugs can be tested, such as antibodies or small inhibitors. Therefore, other than bridging the gap between in vivo and in vitro studies, the use of spheroids can accelerate the setting of protocols for a more personalized medicine, and for precision diagnostics and therapy.
